# Astrocytic C–X–C motif chemokine ligand-1 mediates β-amyloid-induced synaptotoxicity

**DOI:** 10.1186/s12974-021-02371-0

**Published:** 2021-12-28

**Authors:** Beatriz G. Perez-Nievas, Louisa Johnson, Paula Beltran-Lobo, Martina M. Hughes, Luciana Gammallieri, Francesca Tarsitano, Monika A. Myszczynska, Irina Vazquez-Villasenor, Maria Jimenez-Sanchez, Claire Troakes, Stephen B. Wharton, Laura Ferraiuolo, Wendy Noble

**Affiliations:** 1 Department of Basic and Clinical Neuroscience, Maurice Wohl Clinical Neuroscience Institute, Institute of Psychiatry, Psychology and Neuroscience, King’s College London, 5 Cutcombe Road, London, SE5 9RX UK; 2 Sheffield Institute for Translational Neuroscience (SITraN), University of Sheffield, Sheffield, S10 2HQ UK

**Keywords:** Astrocyte, Synapse, Beta-amyloid, Alzheimer’s disease, CXCL1, Tau

## Abstract

**Background:**

Pathological interactions between β-amyloid (Aβ) and tau drive synapse loss and cognitive decline in Alzheimer’s disease (AD). Reactive astrocytes, displaying altered functions, are also a prominent feature of AD brain. This large and heterogeneous population of cells are increasingly recognised as contributing to early phases of disease. However, the contribution of astrocytes to Aβ-induced synaptotoxicity in AD is not well understood.

**Methods:**

We stimulated mouse and human astrocytes with conditioned medium containing concentrations and species of human Aβ that mimic those in human AD brain. Medium from stimulated astrocytes was collected and immunodepleted of Aβ before being added to naïve rodent or human neuron cultures. A cytokine, identified in unbiased screens of stimulated astrocyte media and in postmortem human AD brain lysates was also applied to neurons, including those pre-treated with a chemokine receptor antagonist. Tau mislocalisation, synaptic markers and dendritic spine numbers were measured in cultured neurons and organotypic brain slice cultures.

**Results:**

We found that conditioned medium from stimulated astrocytes induces exaggerated synaptotoxicity that is recapitulated following spiking of neuron culture medium with recombinant C–X–C motif chemokine ligand-1 (CXCL1), a chemokine upregulated in AD brain. Antagonism of neuronal C–X–C motif chemokine receptor 2 (CXCR2) prevented synaptotoxicity in response to CXCL1 and Aβ-stimulated astrocyte secretions.

**Conclusions:**

Our data indicate that astrocytes exacerbate the synaptotoxic effects of Aβ via interactions of astrocytic CXCL1 and neuronal CXCR2 receptors, highlighting this chemokine–receptor pair as a novel target for therapeutic intervention in AD.

**Supplementary Information:**

The online version contains supplementary material available at 10.1186/s12974-021-02371-0.

## Background

Synapse loss in neocortex and limbic areas is an early pathological feature of Alzheimer’s disease (AD) that correlates strongly with cognitive decline [1, 2]. The extent of synapse loss cannot be fully accounted for by loss of neurons alone, implying that surviving neurons also lose synapses [3, 4]. Loss of connectivity between surviving neurons in AD brain damages the efficiency of neural systems, explaining the association between synapse loss and cognitive decline.

Bioactive soluble dimers, oligomers and to a lesser extent N-terminally extended Aβ peptides produced by cultured cells, transgenic rodent models of AD, or extracted from human AD brain, reduce dendritic spine number and disrupt synaptic function, long-term potentiation and rodent cognition [5–9]. Yet, it remains to be established whether clearance of Aβ alone is sufficient to prevent synaptic degeneration in AD [10]. Missorting of modified forms of tau to both the pre-synapse and dendritic spines is also linked with synaptotoxicity in AD [11–14]. We previously reported an association of synaptic tau with dementia in AD. Phosphorylated and oligomeric forms of tau were redistributed from the cytosolic compartment into synaptoneurosomes in cases with typical AD pathology, synapse loss and dementia, but not in so-called “mismatches” who showed a similar burden of AD pathology but preserved synaptic protein levels and cognition [15]. In addition, we saw increased astrocyte reactivity, as indicated with glial fibrillary acidic protein (GFAP) immunolabelling, in those with AD and dementia relative to mismatch cases [15].

Astrocytes are an intrinsic component of synapses [16] and affect synaptic activity by regulating the availability of glutamate, gamma-aminobutyric acid (GABA), adenosine triphosphate (ATP), and glucose [17–20]. As such, healthy functioning astrocytes are critical for synaptic transmission [21], neural circuit maintenance [22] and long-term potentiation [23].

In AD, and particularly in association with elevated Aβ or amyloid plaques [24–26], astrocytes become “reactive” leading to changes in their morphology, molecular fingerprint and function [27, 28], and reactive astrocytes are often closely associated with increased disease severity and cognitive decline [29].

However, the specific contribution of reactive astrocytes to AD pathogenesis remains unclear, with some suggesting that reactive astrocytes lose synaptic and neuronal support functions [30] or that astrocytes undergo cellular senescence in AD [31]. Others report neurotoxic effects of reactive astrogliosis, including those mediated by inflammatory astrocytic secretions [32–34]. Particularly in response to repeated insult, systemic or secondary inflammation, astrocytes show exaggerated production and secretion of pro-inflammatory cytokines including interleukin (IL)-1beta, IL-6 and chemokines, such as CXCL1 [35, 36].

Reactive astrocytes may also be neuroprotective, with recent reports showing that astrocytic IL-3 signals to microglia to promote their phagocytosis of aggregated tau and Aβ [37]. This divergence in astrocytic response may be at least partly related to astrocytic heterogeneity in different brain regions and in response to different types of acute injury [30, 38, 39]. Here, we sought to better understand the contribution of astrocytes to synaptotoxic Aβ-tau interactions in AD by exposing rodent and human astrocytes to concentrations and species of human Aβ that replicate those found in human AD brain.

## Methods

### Postmortem human brain

Post-mortem human prefrontal cortex (Brodmann area 9; BA9) from control and clinically and pathologically confirmed cases of Alzheimer’s disease were obtained from the Neurodegenerative Diseases Brain Bank, King’s College London. Samples were collected from control cases (Braak stage 0, *n* = 4) and those with mild AD neuropathology (Braak stages I–II, *n* = 7), moderate AD neuropathology (Braak stages III–IV, *n* = 10), and severe AD neuropathology (Braak stages V–VI, *n* = 6) (Table 1). There were no significant differences in age, gender or post-mortem delay between groups.

**Table 1:** Human postmortem samples from BA9 prefrontal cortex, that were used in this study

Sex	Age (Y)	PMD (h)	Braak stage	ApoE	Observations
M	59	50	0	n/a	Normal adult brain
M	40	40	0	n/a	Normal adult brain
M	78	10	0	2/3	Ageing changes (mild)
F	82	13	0	2/3	Argyrophilic grains low to moderate density
F	92	17	I	3/3	Control brain with some tau deposition
F	55	12	I	n/a	Minimal tau pathology consistent with HP-tau stage-I
F	81	17	I	3/3	Mild ageing changes Braak I
M	81	18	I	n/a	control—old cerebral infarct (Braak I)
M	93	33	II	3/3	Mild alzheimer-type changes—Braak II
F	84	35	II	n/a	Alzheimer changes Braak II consistent with normal ageing
F	97	39	II	n/a	Very mild Alzheimer’s disease type changes BNE stage 2, control
M	92	11	III	n/a	Mild alzheimer-type changes—Braak III
F	70	38	III	n/a	Possible AD (CERAD) Braak III (limbic) BNE stage III
M	86	52	III	n/a	Alzheimer type changes (ageing), BNE stage 2, control
F	98	3.5	III	n/a	Alzheimer’s disease BNE IV, CERAD probable; TDP-43 pathology (hippocampus and amygdala)
F	89	36.5	III	3/3	Alzheimer’s disease pathology (BNE stage IV) with amyloid angiopathy & limbic TDP-43 pathology but cognitively not impaired
M	82	28	IV	n/a	AD (modified Braak IV) with limbic TDP-43 pathology
M	86	53	IV	n/a	AD (modified Braak IV) with extensive severe amyloid angiopathy
F	83	22	IV	n/a	AD (limbic stage-modified Braak IV) with moderate–severe amyloid angiopathy
F	89	56	IV	n/a	AD HP tau stage 4, severely affecting limbic system and moderately extending to neocortex
M	92	70	IV	n/a	Alzheimer’s disease (BNE stage 4)
F	80	13	V	3/4	AD Braak V with mild amyloid angiopathy
M	86	26	V	4/4	AD Braak V with moderate amyloid angiopathy
F	84	24	V	4/4	Alzheimer’s disease Braak 5
F	97	12	V	3/3	Alzheimer’s disease Braak V
M	72	5	VI	3/3	AD Braak VI with marked amyloid angiopathy
F	91	28.5	VI	3/4	Alzheimer’s disease, Braak VI

Age is shown in years (y), postmortem delay (PMD) in hours (h), sex, ApoE genotype (when known) and Braak stage. Braak stage 0 cases had some phospho-tau but did not meet Braak stage I criteria and were used as controls

### Animals

All animal work was conducted in accordance with the UK Animals (Scientific Procedures) Act 1986 and the European Directive 2010/63/EU under UK Home Office Personal and Project Licenses and with agreement from the King’s College London (Denmark Hill) Animal Welfare and Ethical Review Board. Pregnant female CD1 mice purchased from Charles River were used to prepare mouse primary cultures and organotypic brain slices within 5 days of delivery. Tg2576 mice were used to prepare primary neurons that secrete human Aβ. Tg2576 mice overexpress human APP (isoform 695) containing the double mutation K670N, M671L (Swedish mutation) under the control of the hamster prion protein promoter [40]. Tg2576 mice were originally obtained from Taconic farms (Germantown, NY, USA), and were maintained in-house by breeding males with C57Bl/6/SJL F1 females as recommended by the suppliers. The genotype of the animals was determined by polymerase chain reaction on DNA obtained from the embryos, as previously described [41]. Water and food were available (Picolab rodent diet 20; # 5053; Lab Diet, St Louis, MO, USA) ad libitum. Animals were housed at 19–22 °C, humidity 55%, 12-h:12-h light:dark cycle with lights on at 07:30.

### Mouse primary neural cell cultures

To obtain Aβ-containing medium (transgenic conditioned media or TGCM), we prepared primary neurons from the cerebral cortex of Tg2576 mice at embryonic day 15, as previously described [42]. Neurons were plated at a density of 6 × 10^5^ viable cells/35mm^2^ on glass-bottomed dishes (MatTek Corporation, Ashland, MA, USA), 96-well plates (BD Falcon) or 12-well Nunc™ plates (ThermoFisher Scientific) previously coated with PDL (10 μg/ml) for at least 1 h at 37 °C. Cultures were maintained at 37 °C in 5% CO_2_, in neurobasal medium with 2% B27 nutrient, Glutamax, penicillin (100 units/ml) and streptomycin (100 μg/ml). After 14 DIV medium from healthy neurons was collected [43]. Media collected from 14 DIV wild-type neurons cultured from littermates (wild type conditioned media or WTCM) was used as a control.

Primary neurons were obtained from cerebral cortex of CD1 embryos as described above. Neurons were transfected at 5–7 DIV with the plasmid peGFP-N1 (Clontech, Mountain View, CA) using lipofectamine 2000 (ThermoFisher Scientific).

Primary astrocytic cultures were prepared from the cortex of wild type CD1 mice on postnatal days 1–3 as previously described [44]. The astrocytes were seeded onto a poly-d-lysine (PDL 10 μg/ml) precoated T75 flasks and maintained in culture for 7–10 days in a humidified CO_2_ incubator at 37 °C with shaking at 200 rpm overnight on days 3 and 7 to remove remaining microglia and oligodendrocytes. Astrocyte-enriched cultures were trypsinized using TrypLE (ThermoFisher Scientific) and replated on PDL precoated 6- or 12-wells plates until they reached 13–14 DIV. 24 h before treatment (as described below), growth medium (high glucose DMEM with glutamax, 10% fetal bovine serum, 100 units/ml penicillin and 100 μg/ml streptomycin) was changed to Neurobasal-B27 serum-free medium.

For co-culture experiments, primary astrocytes were plated on cell-culture inserts (0.4 µm pore membrane, Falcon, Corning, Corning, NY, USA) that allow the passage of small molecules between cells and culture medium, and neurons were plated on 6 well plates, as described above. Astrocytes were treated with TGCM or WTCM for 24 h, the medium was removed (TGCM astro and WTCM astro), the cells washed with PBS and inserts placed on top of cultured neurons for another 24 h. There was no direct contact between neurons and astrocytes.

### Human astrocyte and neuronal cell cultures

Skin fibroblasts from three control subjects were used (Table 2). These were reprogrammed as previously described [45]. Once induced, neuronal progenitor cells (iNPC) cultures were established, and the medium was switched to NPC proliferation media consisting of DMEM/F12 (1:1) GlutaMax, 1% N2, 1% B27, and 40 ng/ml FGF2. iNPC-derived astrocytes were produced as previously described [45–47]. Briefly, iNPCs were switched to astrocyte proliferation media, DMEM (Thermo Fisher Scientific), 10% FBS (Life science production, Bedford, UK), 0.2% N2 (Thermo Fisher Scientific). Cells were differentiated in 6-well plates coated with human fibronectin for 7 days and were used at a matched number of passages.

**Table 2:** Characteristics of human donors from whom fibroblasts were obtained

Line ID	Type	Sex	Age	Ethnicity
155	Control	M	40	Caucasian
209	Control	F	69	Caucasian
3050	Control	M	65	Caucasian

Line ID, type (disease or control), sex, age in years and ethnicity are shown

Lund human mesencephalic (LUHMES) neuronal precursors were grown in T75 flasks in proliferation medium DMEM/F12 GlutaMAX™ supplement medium (Thermo Fisher Scientific), N2 supplement (Thermo Fisher Scientific) and 40 ng/ml recombinant basic fibroblast growth factor (FGF) (Peprotech). To allow high content imaging on the Opera Phenix microscope, non-differentiated LUHMES were transduced with GFP-expressing lentiviral particles (LV-GFP), with GFP expression under the control of a PGK promoter as previously described [48]. When cells were 50–60% confluent they were differentiated by adding differentiation media consisting of DMEM/F12 GlutaMAX™ supplement medium, N2 supplement and 1 μg/ml tetracycline. After 2 days, LUHMES were trypsinized and replated onto 96 wells plates for a further 3 days before experimentation.

### Mouse organotypic brain slice culture

Organotypic brain slice cultures were prepared from P9 wild-type CD1 mice and cultured as described in Croft et al. [49]. Briefly, pups were culled by decapitation in accordance with the UK Animals in Scientific Procedures Act (1986). Brains from pups were bisected into hemi-brains by a single cut along the midline. The cerebellum, thalamus and brainstem were removed and discarded to leave the cortex, hippocampus and connecting areas. These were kept in ice-cold dissection buffer [1.25 mM KH_2_PO_4_ pH 7.4, 124 mM NaCl, 3 mM KCl, 8.19 mM MgSO_4_, 2.65 mM CaCl_2_, 3.5 mM NaHCO_3_, 10 mM glucose, 2 mM ascorbic acid, 39.4 µM ATP in ultrapure H2O, sterile filtered (0.2 µm)] with constant oxygenation throughout the preparation procedure. 350 µm coronal slices were cut using a McIlwain Tissue Chopper (Stoelting Europe, Ireland). Eighteen slices from each hemi-brain were collected and 3 consecutive slices per well were positioned on interface-style Millicell culture inserts (Millipore (UK) Ltd.) in 6 well culture plates (ThermoFisher Scientific, UK) containing 1 mL of sterile slice culture medium (Basal medium eagle (BME), 1 mM HEPES, pH 7.1, 19.3 mM NaCl, 5 mM NaHCO_3_, 511 µM ascorbic acid, 40 mM glucose, 2.7 mM CaCl_2_, 2.5 mM MgSO_4_, 1% (v/v) GlutaMAX (Life Technologies, Paisley, UK), 0.033% (v/v) insulin, 0.5% (v/v) penicillin/streptomycin (Life Technologies), in ultrapure H_2_O, sterile filtered (0.2 µm), plus 25% (v/v) heat inactivated horse serum (ThermoFisher, UK). Three hours after plating, the culture medium was removed by aspiration and replaced with 1 ml of pre-warmed fresh sterile culture medium. Brain slices were incubated at 37 °C and the culture medium was changed from the bottom of each well every 2–3 days. Slices were maintained for a minimum of 14 day in vitro prior to treatment with WTCM or TGCM as described below.

### Cell and slice culture treatments

To treat cells and slice cultures with concentrations and species of Aβ that mimic those in human AD brain, we collected the medium from cultured Tg2576 neurons. Aβ in this culture medium is naturally secreted at a 10:1 ratio of Aβ40:Aβ42. The concentration of Aβ in TGCM was determined by ELISA (ThermoFisher Scientific) following the manufacturer’s instructions. To mimic Aβ levels in human AD brain [50], medium was diluted (in culture medium) to reach 2000 pM Aβ40/200 pM Aβ42, as described previously [42, 50–52]. An equivalent volume of WTCM was used as control. TGCM or WTCM was added directly to the culture medium of mouse or human astrocyte or neuron cultures, or directly on top of mouse organotypic brain slice cultures, for 24 or 48 h.

To test the direct effects of the cytokine CXCL1 on synapse/neuronal health, DIV12–13 neurons were pre-treated for 20 min with 40 ng/ml of an antagonist of the CXCL1 receptor in neurons, CXCR2 (SB225002, Tocris Bioscience), prior to the addition of 40 nM recombinant mouse CXCL1 (R&D Systems) or vehicle for a further 24 h.

### Immunodepletion of Aβ

To remove Aβ from the medium of astrocytes challenged with TGCM prior to application of astrocyte conditioned medium to neurons, immunodepletion was used, as previously described [50]. Briefly, 24 h after treatment of astrocytes with TGCM, medium was collected and incubated with protein G Dynabeads (ThermoFisher) bound with 9 μg of 6E10 antibody (COVANCE) for 90 min at 4 °C, according to the manufacturer and previously published protocols [50]. Beads were separated from medium using a magnetic stand to remove Aβ-6E10 complexes. The efficacy of immunodepletion was assessed by measuring levels of Aβ before and after 6E10 immunodepletion by ELISA and western blot (Additional file 1: Fig. S1A, B).

### Isolation of synaptoneurosomes and analysis of protein content in each fraction

Total, cytosolic and synaptic fractions were isolated from human brain tissues or mouse organotypic brain slice cultures as previously described [15, 53]. Briefly, synaptoneurosomes were prepared from ∼ 250 mg of frozen tissue (grey matter) or from three pooled wells (9 consecutive slices) of slice cultures. Tissue was homogenized in 1.5 ml or 250 μl of ice-cold Buffer A (25 mM HEPES, pH 7.9, 120 mM NaCl, 5 mM KCl, 1 mM MgCl_2_, 2 mM CaCl_2_, 1 mM dithiothreitol, protease inhibitors, phosphatase inhibitors) for brain tissue or slices, respectively, using a Teflon-glass mechanical tissue grinder at 170 rpm and filtered through 80 µm pore filters. A portion of the filtrate was collected, supplemented with 1.5% SDS, boiled for 5 min, and centrifuged at 15 000 *g* for 15 min, to give the total protein fraction. The remaining sample was filtered through 5 µm pore filters and centrifuged at 1000 *g* for 10 min to pellet synaptoneurosomes. The supernatant was collected as cytosolic extract, which was further centrifuged at 100, 000 *g* for 30 min to remove microsomes. The synaptoneurosome pellet was washed once with cold Buffer A and centrifuged again at 1000 *g* for 10 min. The pellet was extracted with Buffer B (50 mM Tris pH 7.5, 1.5% SDS, 2 mM dithiothreitol) (0.5 ml for human brain, 80 μl for slices) and boiled for 5 min. After centrifugation at 15,000 g for 15 min, the supernatant was collected as the synaptoneurosome fraction.

Protein concentrations in each fraction were determined using a bicinchoninic acid (BCA) protein concentration assay kit (Pierce) according to the manufacturer's instructions and were adjusted to the same concentration for all fractions by adding homogenisation buffer*.* Tau content in total, cytosolic and synaptoneurosomal fractions was examined for organotypic brain slice culture experiments. The cytosolic fraction of postmortem human brain was used for cytokine arrays.

### Cytokine arrays

The cytoplasmic fraction of human postmortem brain homogenates and astrocyte culture media were used to determine cytokine content using Mouse or Human Proteome Profiler arrays (Mouse Cytokine Array Panel A and Human Cytokine Array, R&D Systems), according to the manufacturers’ instructions. Positive and negative control spots included were used to allow quantitative analysis of cytokine levels. Results were expressed as percentage change compared to controls.

### SDS-PAGE and western blotting

To collect a total protein fraction of cultured cells for SDS-PAGE and western blot, cultured cells were washed with PBS to remove medium and were directly lysed into PBS containing sample buffer (NuPAGE LDS Sample Buffer 4X, Invitrogen), reducing agent (NuPAGE Sample Reducing Agent 10X, Invitrogen), protease inhibitor (complete Mini EDTA-Free Protease Inhibitor Cocktail, Roche, Basel, CH) and phosphatase inhibitor (PhosSTOP, Roche) cocktails.

Equal protein amounts were separated on 10% or 4–12% SDS-PAGE gels (Thermo Fisher Scientific) by electrophoresis, transferred to 0.45 μm nitrocellulose membranes (Millipore, MA, USA), and immunoblotted as described previously (Glennon et al. 2020). Primary antibodies were against PSD-95 (Rabbit IgG, #3409, Cell Signalling 1/500), synapsin-1 (Rabbit IgG, #BV-6008, Enzo 1/500), caspase-3 (Rabbit IgG, #9661, Cell Signalling 1/500), GFAP (Mouse IgG, Z0334, DAKO Ltd, 1/500), phosphoNFkB (Rabbit IgG, #3031, Cell Signalling, 1/1000), β-amyloid-6E10 (Mouse IgG, SIG-39320, 1/200–1/500), lipocalin-2/NGAL (Mouse IgG, #AF1857, 1/500), tau (Rabbit IgG, #A0024, DAKO Ltd, 1/5000), PHF1 (Mouse IgG, P. Davies, 1/2000), GAPDH (Mouse IgG, #32233, SantaCruz Biotechnology, 1/500), β-actin (Mouse IgG, #ab8226, 1/1000), α-tubulin (Rabbit IgG, #ab18251, 1/1000). Bound antibodies were imaged using an Odyssey CLX instrument (LI-COR Biosciences) and band intensities were quantified using the Image Studio Software (LI-COR Biosciences). For analysis of synaptic proteins and markers of astrocyte reactivity, the band intensity of the protein of interest was normalised to that of a loading control (β-actin or GAPDH) in the same sample. For western blots of synaptoneurosome, the amount of total tau or phosphorylated tau in the synaptoneurosome fraction was normalised to the amount of total tau or phosphorylated tau in the cytosolic fraction of the same sample. Data is shown as % average control, with the control for each experiment stated in the figure legend.

### Immunocytochemistry

Cells were fixed for 10 min in 4% paraformaldehyde and 4% sucrose in PBS and then for 10 min in pre-chilled methanol. Cells were simultaneously permeabilized and blocked using PBS containing 2% normal goat serum and 0.1% Triton-X-100 for 1 h at room temperature. Immunolabelling was performed according to previously published protocols [54]. Primary antibodies were against tau (Rabbit IgG, #A0024, DAKO, 1/500), MAP2 (Mouse IgG, #GTX8266, GeneTex, 1/200), and GFAP (Rabbit IgG, #Z0334, DAKO, 1/100), and the appropriate species of AlexaFluor-conjugated secondary antibodies (Goat anti-rabbit IgG or Goat anti-mouse IgG, Life Technologies). High-resolution digital images were acquired using the Opera Phenix High Content Screening System in confocal mode with a Zeiss 20 × water NA 1.0 objective. The fluorophores were detected with the following excitation and emission (Ex/Em) wavelengths: Hoechst 33342 (405/435–480), AF488 (488/500–550), and far red (wavelengths above 655 nm). Cell segmentation and quantification analyses were performed using the Harmony software, version 4.9. The experimenter was blinded to treatment.

### Measurement of dendritic spine density, neuron complexity and tau mislocalisation

For the analysis of dendritic spine structure and neuronal complexity, neurons at 14 DIV were transfected with the plasmid peGFP-N1 (Clontech, Mountain View, CA) using Lipofectamine 2000, fixed 24 h post-transfection, and the GFP-expressing cells were imaged using high-resolution confocal digital images obtained from an Opera-Phenix microscope (Perkin-Elmer) as described above. NeuronStudio software (CNIC, Mount Sinai School of Medicine) was used for dendritic spine analysis. Dendritic spine densities were calculated from approximately 20 neurons/condition for each biological replicate. Spine density, or number of neuritic protrusions was defined as number per micrometer of dendrite/neurite length. The experimenter was blinded to treatment.

Neuronal complexity was analysed as a measure of neuron health using Harmony software which gives automated (therefore, blinded) measures of mean and maximum neurite length, number of roots (number of junctions of neurites with the cell body), nodes (number of neurite branch points), segments (total number of individual branches) and extremities (number of distinct neurite ends). A reduction in neurite number or in complexity measures is indicative of reduced neuronal health [34]. The number of neurites per cell containing missorted tau was assessed with Harmony™ software using an algorithm that identifies neurite segments (labelled with MAP2) that contain tau.

### Statistical analysis

Data were analysed using GraphPad Prism. After performing a Shapiro–Wilk normality test, most data were analysed using one-way ANOVA followed by Tukey post hoc test or Student’s *t* test (GraphPad Prism 7 Software, Graphpad Software, La Jolla, CA, USA). Results were considered statistically significant when *p* < 0.05. For neurons treated with astrocyte conditioned media and the CXCR2 antagonist, a two-way ANOVA followed by post hoc tests was performed (see figure legends). Data are shown as mean ± standard error of the mean (SEM).

## Results

### Conditioned medium from Aβ stimulated astrocytes is synaptotoxic

**Fig. 1:**
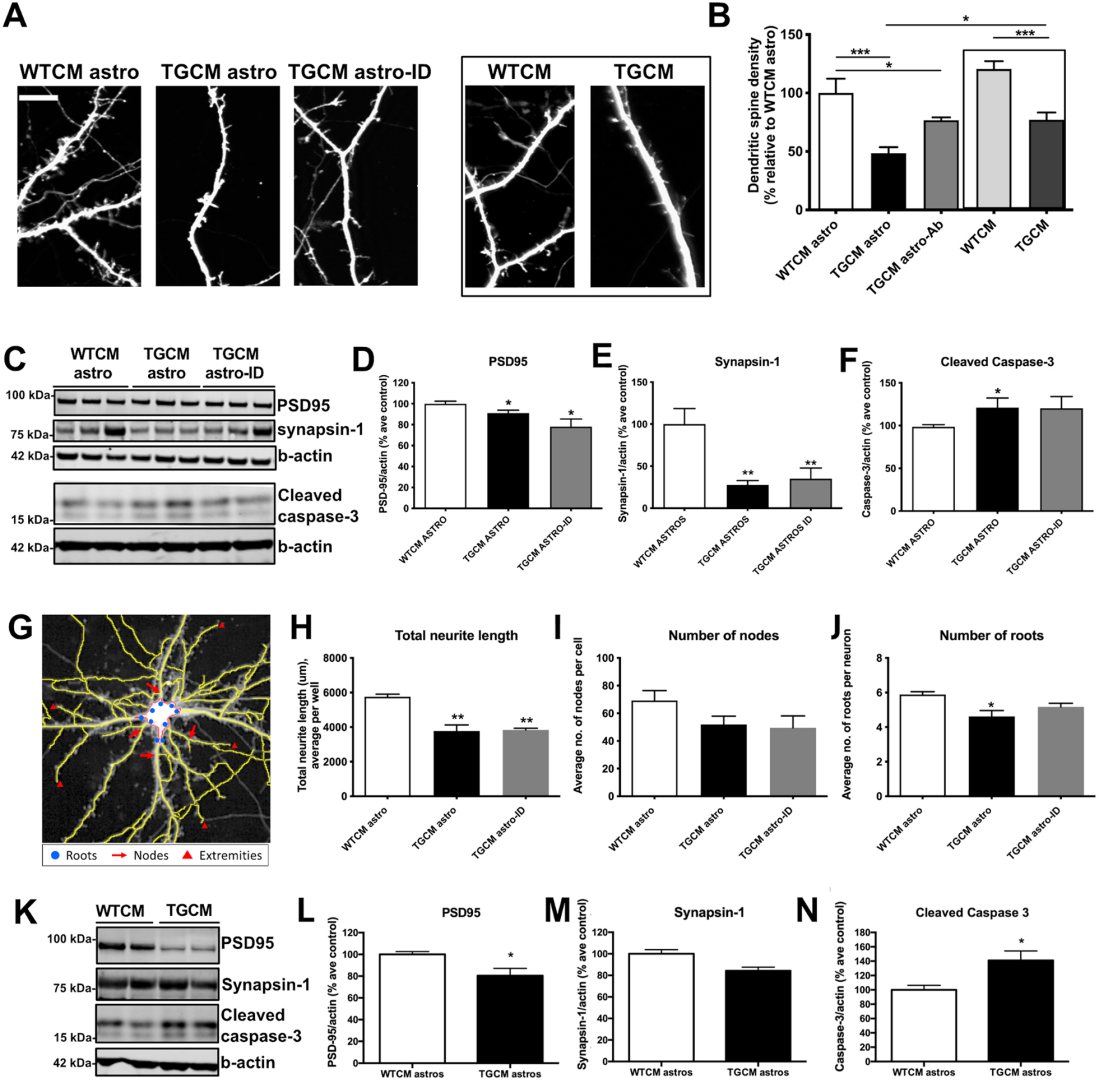
Conditioned medium from Aβ-stimulated astrocytes exaggerates the damaging effects of Aβ at synapses. **A** Dendritic spine density was measured in neurons transfected at 7DIV with GFP and treated with WTCM astro, TGCM astro and TGCM astro-ID or directly with WTCM and TGCM at 13DIV for 24 h. Representative images are shown. Scale bar is 10 μm. **B** Quantification of spine density in 10–20 neurons per experimental replicate from eight individual neuron preps (*n* = 8). TGCM astro caused increased dendritic spine loss relative to treatment with TGCM directly, and this is maintained when Aβ is immunodepleted from the medium. Box indicates data from neurons directly exposed to TGCM or WTCM for clarity. Data was analysed by ordinary one-way ANOVA and Tukey’s post-hoc tests. **C** Neuronal lysates were immunoblotted using antibodies against PSD-95 (95 kDa), synapsin-1 (77 kDa), cleaved caspase-3 (17–19 kDa) and β-actin (42 kDa). Representative blots are shown. Western blot band intensities were quantified and showed that TGCM astro caused reductions in the abundance of the synaptic markers **D** PSD-95 and **E** synapsin-1 and an increase in **F** cleaved (active) caspase-3, that is maintained when Aβ was immunodepleted (TGCM astro-ID). Data is from eight individual neuron preps (*n* = 8), each of which have two experimental replicates. Data was analysed by ordinary one-way ANOVA and Tukey’s multiple comparison post-hoc tests. **G** Neuronal complexity as a measure of neuron health was analysed for all cells in three wells of five biological replicates (*n* = 5) using Harmony software. This showed **H** reduced total neurite length, and fewer number of **I** nodes and **J** roots following treatment with TGCM astro and TGCM astro-ID, indicating that astrocytic secretions are damaging to neurons. Data was analysed by ordinary one-way ANOVA and Tukey’s multiple comparison post-hoc tests. **K** Astrocytes were cultured and treated in cell culture inserts, the medium removed by washing and the astrocytes added to neuron cultures. Representative western blots of neuronal lysate from co-culture experiments are shown. Western blot data was quantified by normalising the protein of interest to β-actin levels in the same sample and showed reduced **L** PSD-95 and **M** synapsin-1, and **N** increased levels of cleaved caspase-3 when neurons were co-cultured with astrocytes that had previously been exposed to TGCM (*n* = 6, data is from six independent experiments, each of which had three technical replicates). Data was analysed using an unpaired *t* test. Data on graphs is mean ± SEM and is shown as percentage average control (WTCM astro). *p < 0.05, **p < 0.01

Primary neurons cultured from Tg2576 mice release human Aβ into the culture medium in an approximately 1:10 ratio of Aβ42:Aβ40 as in human AD brain (Wu et al., 2010). Here, conditioned medium was collected from primary cortical neurons from wild-type or Tg2576 mice (WTCM; TGCM), diluted in neurobasal medium to human brain levels (200 pM Aβ42, 2000 pM Aβ40; [50]) and applied to astrocytes or neurons. The conditioned medium from stimulated astrocytes was collected, (WTCM astro and TGCM astro) and in some experiments Aβ was immunodepleted from the medium of TGCM treated astrocytes (TGCM astro-ID). These media were added to naïve WT primary neurons. Direct exposure of naïve neurons to TGCM induces a decrease in the number of dendritic spines compared to neurons treated with medium from wild type littermates (WTCM) [42, 51, 52]. This finding was reproduced here (Fig. 1A) with spine numbers in TGCM-stimulated neurons being reduced by approximately 36% to 66.42 ± 6.233% relative to WTCM conditions (*p* < 0.001). Notably, spine loss was exacerbated (reduction of 51.60 ± 5.26 SEM% relative to WTCM) when neurons were exposed to medium from astrocytes stimulated with TGCM (TGCM astro) compared to those treated with WTCM (WTCM astro). Importantly, this effect is still observed after immunodepleting Aβ from the medium using 6E10 (TGCM astro-ID) (Fig. 1A, B; Additional file 1: Fig. S1), demonstrating that soluble factors released by astrocytes mediate this heightened synaptotoxicity.

A reduction in the levels of synaptic proteins including PSD95 and synapsin-1 was found in neurons exposed to medium from TGCM-stimulated astrocytes compared to medium from WTCM treated astrocytes (Fig. 1C–E). This was accompanied by increases in levels of cleaved (active) caspase-3 (Fig. 1C, F), an executioner caspase which also plays various non-apoptotic roles in neurons including in proteolysis and modulation of synaptic functions [55]. The changes in synaptic proteins and cleaved caspase-3 are induced by astrocytic secretions, since they are still observed after Aβ is immunodepleted from the astrocyte-conditioned TGCM (TGCM astro-ID; Fig. 1D–F). Please note that the difference in cleaved capase-3 between WTCM and TGCM-ID was not significant, as a result of slightly higher levels of variation in the TGCM-ID data (Mean ± SEM: WTCM astro 100 ± 2.496; TGCM astro 121.2 ± 11.08; TGCM astro ID 120.1 ± 13.86). Similarly, neurons treated with medium from TGCM-stimulated astrocytes showed indicators of poor health shown by reduced neuronal complexity measures (Fig. 1G) including significant reductions in total neurite length and number of roots, alongside non-significant decreases in other parameters that measure neuritic ramification (Fig. 1H, J; Additional file 1: Fig. S2).

To validate this data in more physiological co-culture conditions, astrocytes were grown on cell culture inserts and stimulated with TGCM or WTCM. The culture medium was replaced to remove traces of Aβ and the stimulated astrocytes co-cultured with neurons (Fig. 1K). Under these conditions, astrocytic secretions were also found to disrupt synapses indicated by significantly reduced PSD-95 (*p* < 0.05), apparent (but non-significant) reductions in synapsin-1 levels, and significant increases in cleaved caspase-3 (*p* < 0.05) (Fig. 1K–M) in the absence of elevated lactate dehydrogenase (LDH) release (Additional file 1: Fig. S2B). This confirms that factors released by astrocytes in response to concentrations of human Aβ, similar to that found in AD brain, compromise synaptic and neuronal health without causing overt neurotoxicity.

**Fig. 2:**
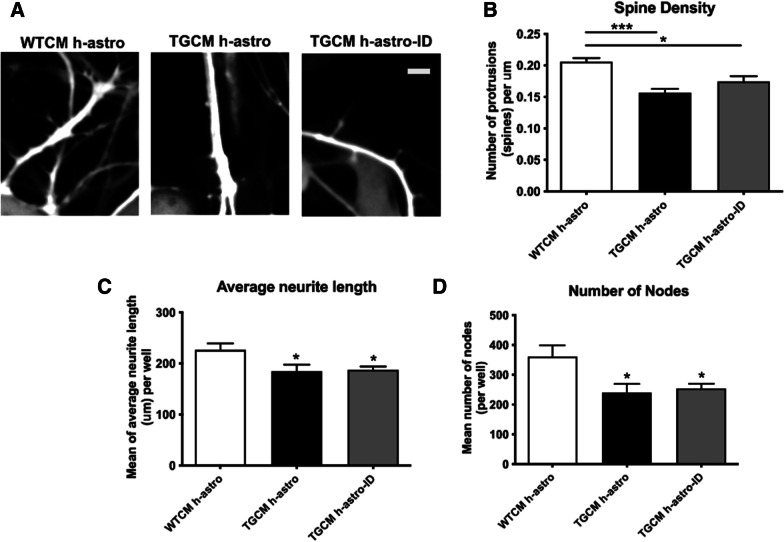
iNPC-astrocytes stimulated with human Aβ release synaptotoxic secretions. Human iNPC-astrocytes were exposed to WTCM and TGCM at 7 day post-differentiation for 24 h. WTCM h-astro and TGCM h-astro was collected and in some cases Aβ was immunodepleted from astrocyte medium (TGCM h-astro-ID). **A** Number of neuritic protrusions (indicated by arrow heads) per μm in LUHMES transfected pre-differentiation GFP were imaged as proxy for dendritic spines in 20 cells per technical triplicate in each of three independent experiments (*n* = 3). Scale bar is 5 μm. **B** Quantification of protrusion number per μm shows reductions in the presence of TGCM h-astro relative to WTCM h-astro that is maintained when Aβ is removed from astrocyte conditioned medium by immunodepletion (TGCM h-astro-ID). Measures of neuron health across three wells for each of three independent experiments (*n* = 3) showed **C** shorter total neurite length and **D** fewer number of nodes in cells with axons following treatment with TGCM h-astro and TGCM h-astro-ID, indicating that human astrocytes secrete factors that are damaging to neurons. Data was analysed by ordinary one-way ANOVA and Tukey’s multiple comparison post-hoc test. Data on graphs is mean ± SEM and is shown relative to control (WTCM h-astro). *p < 0.05, ***p < 0.001

Despite recent studies indicating significant conservation between human and mouse astrocytes, there are species-specific differences in their response to stressors [56]. Therefore, human iNPC-astrocytes, which retain age-related features [57], were stimulated with TGCM or WTCM, the astrocyte conditioned medium collected and immunodepleted of Aβ with 6E10, prior to its addition to human post-mitotic neurons that were differentiated from LUHMES cells. LUHMES are a fetal human mesencephalic cell line conditionally immortalised with a myc transgene which become post-mitotic mature neurons when transgene expression is suppressed [58] and that have utility for high throughput screening of neuronal morphology [59]. Similar to findings with mouse cells, medium from TGCM-exposed human astrocytes (TGCM h-astro) induced a significant loss of neuritic protrusions (Fig. 2A, B, *p* < 0.001) and reductions in features of neuronal health and complexity, measured in cells containing axons, relative to WTCM h-astro (*p* < 0.05, Fig. 2C, D; Additional file 1: Fig. S3), that were retained in the absence of Aβ (TGCM h-astro-ID, *p* < 0.05 for all).

### Astrocyte-mediated synaptotoxicity in response to Aβ is related to tau mislocalisation

**Fig. 3:**
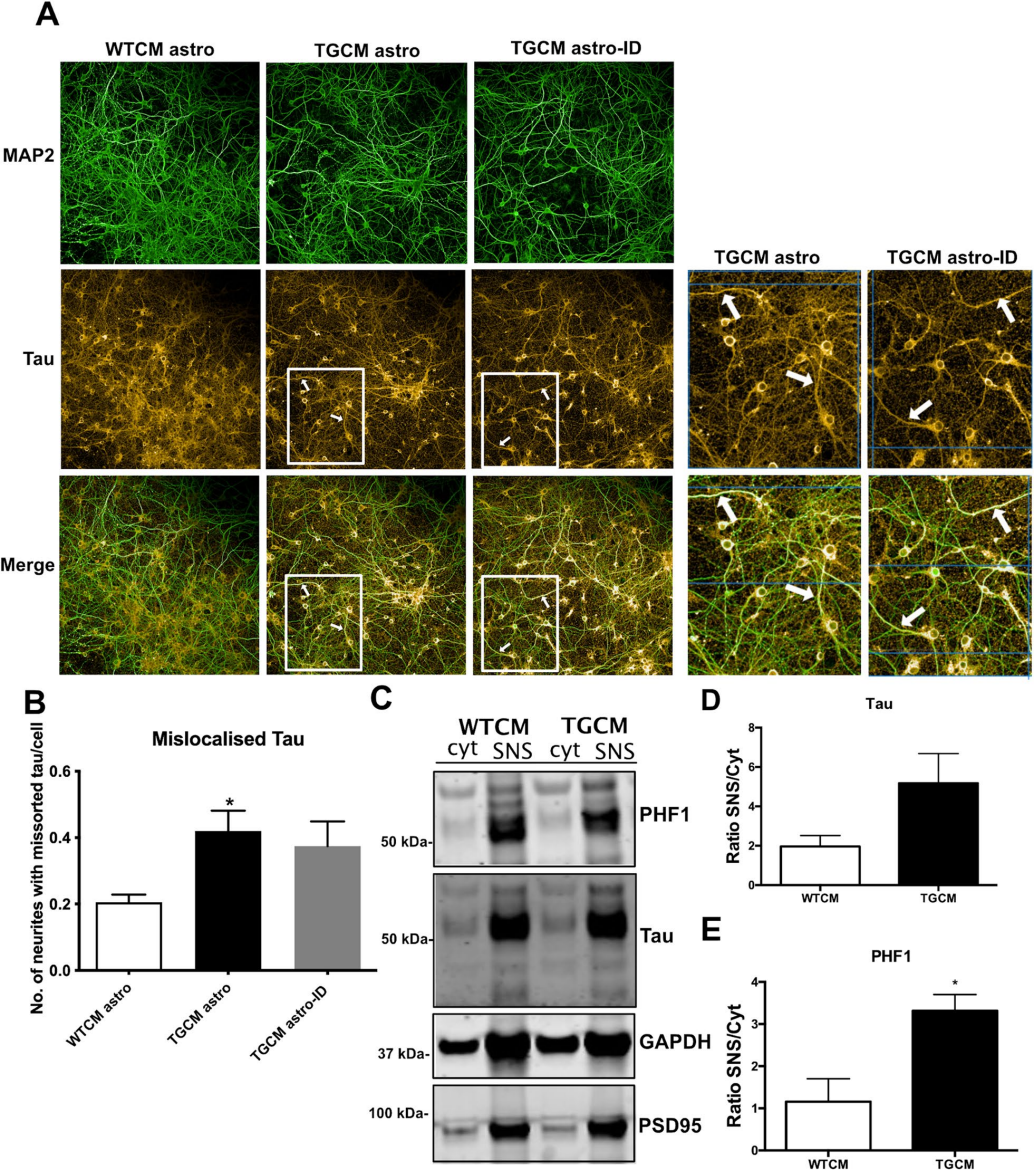
Astrocyte-mediated synaptotoxicity in response to Aβ is accompanied by tau mislocalisation. **A** 14DIV neurons treated for 24 h with WTCM astro, TGCM astro or TGCM astro-ID were fixed and immunolabelled with antibodies against MAP2 (green) and tau (far red). Exposure to TGCM astro and TGCM astro-ID induced increased tau localisation in neurites with white arrows indicating beading of these neurites. Scale bar is 100 μm (20 μm in inset). **B** The number of MAP2 labelled neurites per cell containing missorted tau was quantified, showing increased tau mislocalisation upon exposure to conditioned medium from astrocytes exposed to Aβ (TGCM astro and TGCM astro-ID) (*n* = 4, four independent experiments each performed in triplicate). Data was analysed by ordinary one-way ANOVA and Tukey’s multiple comparison post-hoc test, and is shown relative to control (WTCM astro). **C** To confirm that Aβ induces tau mislocalisation in the presence of other neural cell types, 14DIV organotypic brain slice cultures prepared from CD1 mice were treated with WTCM or TGCM for 24 h. The cytosolic fraction (Cyt) and synaptoneurosomes (SNS) were extracted and immunoblotted with antibodies against PSD-95, total tau and tau phosphorylated at Ser396/404 (PHF1). GAPDH was used as a loading control. PSD-95 accumulates in the synaptic fraction showing successful SNS extraction. The abundance of **D** tau and **E** PHF1 in synaptoneurosomes relative to the amount of tau or PHF1 in the cytosolic fraction of the same sample was determined. Phosphorylated tau showed increased synaptic localisation following exposure to TGCM (*n* = 3, three independent slice culture preparations from which three wells of each were pooled). Data was analysed using unpaired *t* tests. Data on graphs is mean ± SEM and is shown relative to vehicle control. *p < 0.05

Direct effects of Aβ on synapse and neuron health are tau-dependent [11, 57] and related to damaging effects of mislocalised tau at post-synapses [11, 12] and pre-synapses [13, 14]. We found that tau localisation was altered in response to medium from TGCM astro, with tau showing increased neuritic localisation as indicated by elevated numbers of MAP2-positive neurites (green) containing tau (far red) (Fig. 3A, B). Similar findings were observed when organotypic brain slice cultures which contain all neural cell types, were treated with TGCM. Synaptoneurosome fractionations were used to yield cytosolic and synaptic fractions. Western blotting of these samples showed that tau is present in the cytosolic fraction and appears enriched in the synaptic fraction of slices treated with WTCM and TGCM. Quantification of the amount of synaptoneurosome tau relative to tau in the cytosolic fraction of the same sample, as is standard for this type of analysis [15, 53] showed that in slices treated with TGCM the ratio of synaptic to cytosolic tau phosphorylated at Ser396/404 (PHF1) is increased when compared to slices treated with WTCM (Fig. 3C–E).

### Astrocytic inflammatory phenotypes are induced by Aβ

**Fig. 4:**
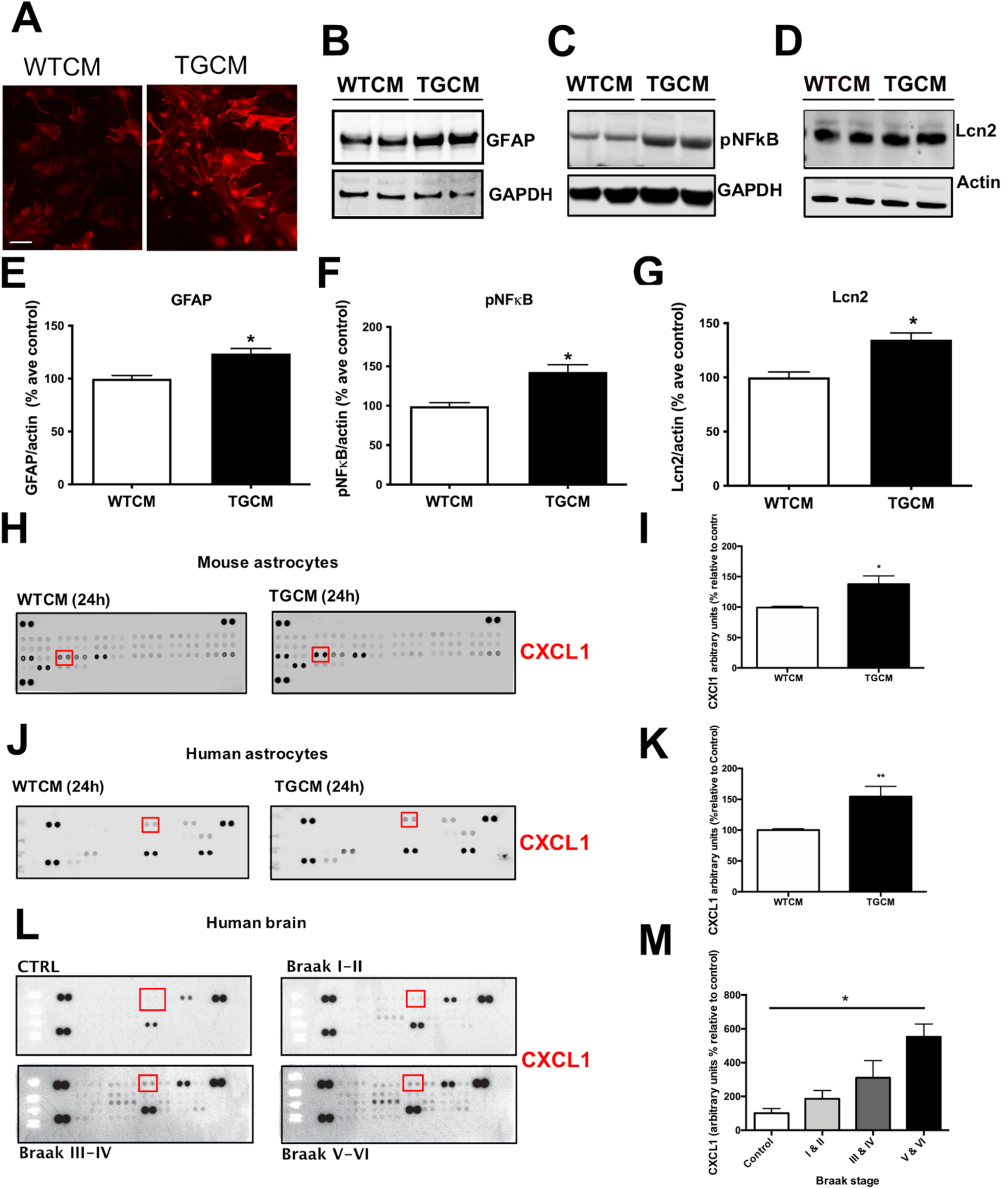
Astrocytic inflammatory phenotypes are induced by Aβ. To explore the effects of physiological Aβ concentrations on astrocyte phenotypes, a number of inflammatory markers were examined. **A** Representative images from mouse astrocytes exposed to TGCM for 24 h showed increased GFAP immunoreactivity (red) relative to those treated with WTCM. Scale bar = 100 μm. Lysates from treated astrocytes were immunoblotted with antibodies against **B** GFAP, **C** phospho-NFkB (p65) and **D** Lcn2. GAPDH was used as a loading control. Quantification of band intensities showed increases in **E** GFAP, **F** pNFkB and **G** Lcn2 in TGCM-treated cultures relative to astrocytes treated with WTCM. Levels of the protein of interest were normalised to GAPDH in each case (*n* = 5, five independent cultures each containing three technical repeats). Data was quantified using unpaired *t* tests. Antibody-based cytokine arrays were used to provide unbiased analysis of cytokine content in medium from WTCM and TGCM exposed mouse and human astrocytes and in tissue homogenates from postmortem AD and control brain. Representative membranes showing increased CXCL1 in TGCM conditions are shown for **H** mouse astrocytes (*n* = 4, four independent experiments each performed in triplicate) and **J** human iNPC-astrocytes (*n* = 3, three independent experiments each performed in triplicate). **L** Total protein normalised cytosolic fractions of postmortem control and BA9 prefrontal cortex of AD brain at different Braak stages (*n* = 4 control, *n* = 7 stage I–II, *n* = 10 stage III–IV, *n* = 6 stage V–VI). Quantification of CXCL1 amounts in these samples revealed significant increases in CXCL1 in TGCM-treated **I**) mouse and **K** human astrocytes, and **M** in Braak stage V–VI human AD brain. Data was analysed using *t* test or one-way ANOVA with Dunnett’s post-hoc tests. Data on graphs is mean ± SEM and is relative to control (control brain or WTCM). *p < 0.05

Although microglia are considered the resident immune cell in the brain, the contribution of astrocytes to neuroinflammatory processes in AD is increasingly recognised [25, 35, 36]. We observed increased levels of GFAP, and other markers of astrocyte reactivity including lipocalin-2 (Lcn2), in astrocytes stimulated with TGCM relative to WTCM (Fig. 4A–G). Lcn2 was identified as a pan-reactive astrocyte marker in gene expression analyses [38] and we previously showed significant elevations in astrocytic lcn2 levels upon their exposure to oxysterol mixtures that mimic their composition in AD brain [34]. These changes reflect the induction of inflammatory signalling pathways in astrocytes, since we observed increased phosphorylation of NFκB (p65) upon exposure of astrocytes to Aβ-containing medium relative to control (WTCM) conditions (Fig. 4C, F). We examined cytokine release from astrocytes using cytokine arrays which allowed unbiased measurement of a panel of cytokines and chemokines in astrocyte conditioned medium. This showed upregulation of a small number of cytokines including CXCL1, M-CSF and CCL2 in medium from TGCM-treated mouse primary astrocytes relative to WTCM treated cells (Fig. 4H, I), and CXCL1 and IP-10 from TGCM-treated iNPC-astro (Fig. 4J, K). Aβ-containing medium significantly increased the secretion of CXCL1 from both mouse and human astrocytes (Fig. 4I, K). Notably, CXCL1 levels are increased in AD brain homogenate relative to samples from age-matched controls (Fig. 4L, M), and CXCL1 has previously been implicated in AD-associated tau changes [60].

### CXCL1 is synaptotoxic

**Fig. 5:**
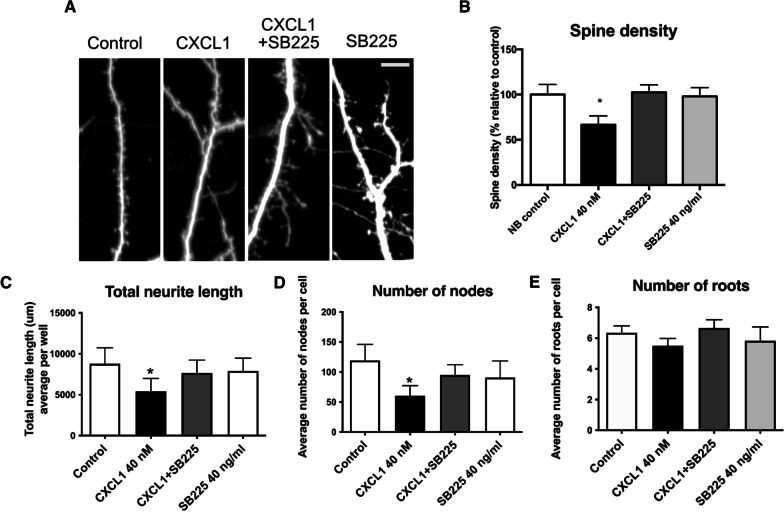
CXCL1 is synaptotoxic. Mouse neurons, transfected at 5-7DIV with GFP, were treated with 40 nM recombinant mouse CXCL1 or vehicle for 24 h to determine if CXCL1 is damaging to neurons. In some cases, neurons were pre-treated for 20 min with 40 ng/ml SB225002, an antagonist of the CXCL1 receptor, CXCR2. Neurons were imaged at 14DIV. **A** Representative images of dendrites in CXCL1 and SB225002 treated conditions (*n* = 3, three independent experiments each with three technical replicates) Scale bar = 10 μm. **B** Quantification of spine density per μm shows reduced spine numbers upon exposure to CXCL1 relative to controls, that was rescued when neurons were pre-treated with the CXCR2 antagonist (*n* = 6, six independent experiments each with three technical replicates). Data was analysed by ordinary one-way ANOVA and Tukey’s multiple comparison post-hoc test. Measures of neuron health across three wells per experimental repeat showed **C** reduced total neurite length, **D** fewer nodes, and **E** fewer roots on exposure of neurons to CXCL1 that is prevented by pre-treatment with SB225002, demonstrating impairments in neuron health as a result of CXCL1 (*n* = 3, three independent experiments each with three technical replicates). Data was analysed by ordinary one-way ANOVA and Tukey’s multiple comparison post-hoc test. Data on graphs is mean ± SEM and is shown relative to control conditions. *p < 0.05

To determine if the synaptotoxic effects of culture medium from TGCM-stimulated astrocytes is mediated by CXCL1, we applied recombinant CXCL1 to primary neurons. This significantly reduced spine number by 66.56 ± 9.89% (Fig. 5A, B), similar to the reductions identified previously using whole TGCM astro. Measures of neuron health including neurite length and number of nodes and roots were also affected by recombinant CXCL1 (Fig. 5C–E). CXCL1-mediated synaptotoxicity was prevented when neurons were pre-treated with an antagonist (SB225) of the CXCL1 receptor, CXCR2 (Fig. 5A–E, Fig. S4). These results strongly implicate CXCL1 in the synaptotoxic effects of astrocytes in response to physiological concentrations of human Aβ.

### CXCL1–CXCR2 interactions mediate the Aβ-induced synaptotoxic responses of astrocytes

**Fig. 6:**
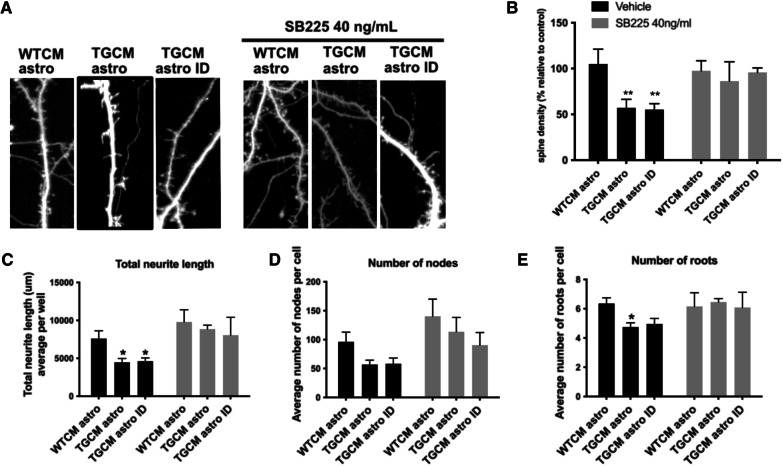
CXCL1–CXCR2 interactions mediate the Aβ-induced synaptotoxic responses of astrocytes. Mouse neurons, transfected at 7DIV with GFP, were exposed to WTCM astro, TGCM astro or TGCM astro-ID for 24 h in the presence or absence of pre-treatment for 20 min with 40 ng/ml SB225002. Neurons were imaged at 14DIV. **A** Representative images of dendritic spines (*n* = 4, four independent experiments each with three technical repeats). Scale bar is 5 μm. **B** Quantification of spine density per μm showed that reductions in spine numbers upon exposure to TGCM astro and TGCM astro-ID is prevented when neurons are pre-treated with the CXCR2 antagonist. Data is normalised to control conditions (WTCM astro, vehicle). Data was analysed using two-way ANOVA with Sidak’s multiple comparisons test. Measures of neuron health across three wells for four independent experiments (*n* = 4) showed **C** reduced total neurite length, **D** fewer nodes, and **E** fewer roots upon exposure of neurons to TGCM astro that is prevented by pre-treatment with SB225002, demonstrating that blocking neuronal CXCR2 prevents the synaptotoxic effects of Aβ-exposed astrocytes. Data was analysed using two-way ANOVA with Sidak’s multiple comparisons test. Data on graphs is mean ± SEM. *p < 0.05, **p < 0.01

Finally, we determined that blocking the CXCR2 receptor is sufficient to prevent synaptotoxicity in response to secretions from Aβ-stimulated astrocytes. Here, astrocytes were stimulated with TGCM or WTCM as before, and the astrocyte conditioned medium was applied to primary neurons that had been pre-incubated with the CXCR2 antagonist SB225. Blocking the CXCL1 receptor prevented the reductions in spine density (Fig. 6A, B) and measures of neuron complexity (Fig. 6C–E) that resulted from Aβ-stimulated astrocyte medium (TGCM astro). These data strongly implicate CXCL1–CXCR2 interactions in the synaptotoxic effects of astrocytes in response to AD-mimicking concentrations of human Aβ.

## Discussion

It is now established that non-neuronal cells, and particularly astrocytes and microglia, make major contributions to the onset and progression of Alzheimer’s disease [61–63]. We add novel data to this growing body of evidence to demonstrate that interactions between the astrocytic chemokine CXCL1 and its neuronal receptor CXCR2 promote synaptotoxicity in the presence of Aβ. As such, our findings further elucidate non-cell autonomous mechanisms underlying synaptotoxic Aβ-tau interactions in AD. There is considerable regional heterogeneity of astrocytes, along with temporal alterations in astrocyte biology with aging and during disease [27, 64] and as such the precise contribution of reactive astrocytes to AD is not clearly established. Our work supports the assertion that rather than becoming reactive secondary to neuronal damage, at least some sub-types of astrocytes respond to AD-mimicking conditions during early “cellular phases” of disease.

Dendritic spines were used here as a measure of synapse health. These structures are post-synaptic sites for the majority of excitatory neurons [65]. Loss of spine density is linked with cognitive decline in humans and is a strong correlate of dementia in AD [1, 66, 67]. We examined dendritic spines in mouse neurons and also neuritic protrusions as a proxy for dendritic spines in human neurons that were differentiated from LUHMES. While LUHMES are a useful model for high throughput screening of changes in neuronal complexity and function [59], our data shows that their protrusion/spine density indicates that they are relatively immature, at least at the time in culture (DIV) examined here. Nevertheless, the changes we showed in the human neurons closely mimicked those we observed in murine primary neurons.

Importantly, reductions in spine density in AD are closely associated with abnormal tau, but less so with Aβ pathology [66], at least in the prefrontal cortex. However, particularly oligomeric forms of Aβ induce network excitability and synaptoxicity in vitro and in vivo [68–71]. Together, these findings suggest that while Aβ may drive synaptic dysfunction in AD, tau is the executioner. Consistent with this assertion, together with a loss of dendritic spines upon exposure to TGCM astro we also see increased neuritic tau that might be indicative of the damaging mis-sorting of tau from the soma to synaptic compartments.

Tau mislocalisation from the cytoplasmic to synaptic fraction of AD brain is closely correlated with dementia in AD [15] and is a key pathological observation in tauopathy brain [72]. Although some tau is found in dendrites under physiological conditions [73, 74], dendritic tau is increased by Aβ, and can interact with post-synaptic components to further mediate excitotoxicity to Aβ [11, 75]. We add to these findings by showing that in addition to having direct effects on neurons, secretions from Aβ-stimulated astrocytes also induce tau mislocalisation and loss of dendritic spines.

“A1” astrocytes were defined by [30] following gene expression analysis from Zamanian et al. [38] as reactive astrocytes with neurotoxic properties. Neurons cultured with “A1” astrocytes, exhibit synaptotoxicity attributed to loss of physiological functions of astrocytes in synaptic maintenance and neuronal homeostasis, alongside secretion of at least one neurotoxic factor [30]. Recently, this group identified saturated lipids contained in Apolipoprotein (APO)E and APOJ lipoparticles as mediators of astrocyte-induced toxicity [76], although it is important to note that this is context-dependent.

Our data show that astrocytes stimulated by Aβ, which is similar in concentration and species to that found in AD brain [42, 51, 52], increase their release of several cytokines, including the inflammatory chemokine CXCL1. Similar findings have been reported in mouse models of AD and prion disease, particularly following a secondary challenge [36]. We provide evidence that CXCL1 is likely one of the neurotoxic factors secreted by astrocytes, since direct application of recombinant CXCL1 recapitulated the loss of dendritic spines that occurs in response to whole conditioned medium from Aβ-stimulated astrocytes (TGCM astro). Importantly, pharmacological blockade of the receptor for CXCL1, CXCR2, prevented both CXCL1- and TGCM astro-induced loss of dendritic spines.

CXCR2 is predominantly expressed in neurons, where it is upregulated proximal to amyloid plaques in AD brain [60]. Interestingly, CXCL1 has previously been shown to induce tau phosphorylation via downstream effects on ERK1/2 and PI-3 kinase [60]. CXCL1 also promotes caspase-3 activation [77]. Caspase-3 activity is increased by Aβ, it cleaves tau into pro-aggregatory fragments that seed neurofibrillary pathology [78] and is closely linked with synaptic disruption in AD [55]. Direct application of CXCL1 to long-term cultured neurons led to caspase-3 activation, caspase-3 mediated tau cleavage, and tau mislocalisation into bead-like varicosities along neuronal processes [77]. Our data, which show that treatment of neurons with TGCM astro, that contains increased levels of CXCL1, led to increased cleaved caspase-3 in neurons and increased levels of neuritic tau, consistent with these previous reports. We speculate that caspase-3 mediated cleavage of tau might induce the mislocalisation of truncated tau into synaptic fractions to disrupt synapse health. It would be interesting to determine if blocking this tau truncation would protect synapses from Aβ-induced toxicity.

## Conclusions

Our data show accelerated loss of dendritic spines upon interactions between CXCL1, secreted from reactive astrocytes in response to TGCM, and neuronal CXCR2 receptors. These data strongly support further investigation of the CXCL1–CXCR2 interaction in rodent models of AD with Aβ (and tau) abnormalities to determine if this ligand–receptor pair is a novel target for therapeutic intervention.

## Supplementary Information

**Additional file 1: Figure S1.** Immunodepletion of Aβ from astrocyte medium with 6E10. **Figure S2.** Measures of neuronal health and complexity in mouse neurons challenged with WTCM astro, TGCM astro and TGCM astro-ID. **Figure S3.** Measures of neuronal health and complexity in LUHMES challenged with WTCM h-astro, TGCM h-astro and TGCM h-astro-ID. **Figure S4.** Immunofluorescence of tau relative to MAP2 in neurons exposed to CXCL1.

## Data Availability

Summary data are available from the authors on request.

## Abbreviations

Aβ: Beta-amyloid
AD: Alzheimer’s disease
ATP: Adenosine triphosphate
APO (E/J): Apolipoprotein
BA: Brodmann area
BCA: Bicinchoninic acid
CCL2: C–C motif chemokine ligand 2
CXCL1: C–X–C motif chemokine ligand 1
CXCR2: CXC chemokine receptor 2
ERK: Extracellular signal related kinase
FGF: Fibroblast growth factor
GABA: Gamma-aminobutyric acid
GFAP: Glial fibrillary acidic protein
IL: Interleukin
IP-10: Interferon gamma-induced protein 10 (CXCL10)
LUHMES: Lund human mesencephalic cells
MAP2: Microtubule associated protein 2
M-CSF: Monocyte colony stimulating factor
PI-3 kinase: Phosphoinositide 3-kinase
PSD-95: Postsynaptic density protein-95
TGCM: Conditioned medium from Tg2576 neurons
TGCM astro: Conditioned medium from astrocytes stimulated with TGCM
TGCM astro ID: Conditioned medium from astrocytes stimulated with TGCM and immunodepleted of Aβ
TGCM h-astro: Conditioned medium from human astrocytes stimulated with TGCM
TGCM h-astro ID: Conditioned medium from human astrocytes stimulated with TGCM and immunodepleted of Aβ
WTCM: Conditioned medium from wild-type neurons
WTCM astro: Conditioned medium from astrocytes stimulated with WTCM
WTCM astro: Conditioned medium from astrocytes stimulated with WTCM
WTCM h-astro: Conditioned medium from human astrocytes stimulated with WTCM
