# Supplementary material for: Astrocytic C–X–C motif chemokine ligand-1 mediates β-amyloid-induced synaptotoxicity
Source: J Neuroinflammation. 2021 Dec 28;18:306. doi: 10.1186/s12974-021-02371-0 (PMC8715604; doi:10.1186/s12974-021-02371-0)
Supplement: Supplementary file 1 — Additional file 1: Figure S1. Immunodepletion of Aβ from astrocyte medium with 6E10. Figure S2. Measures of neuronal health and complexity in mouse neurons challenged with WTCM astro, TGCM astro and TGCM astro-ID. Figure S3. Measures of neuronal health and complexity in LUHMES challenged with WTCM h-astro, TGCM h-astro and TGCM h-astro-ID. Figure S4. Immunofluorescence of tau relative to MAP2 in neurons exposed to CXCL1. [file 12974_2021_2371_MOESM1_ESM.docx]

**Additional data**

**Additional Figure S1**

**A)**


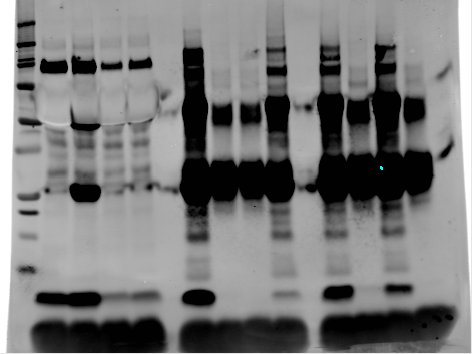


TGCM

TGCM astro (24h)

TGCM+beads+6E10

TGCM astro+beads+6E10

TGCM+beads

TGCM astro+beads

INPUT

IP

250

150

100

75

50

37

25

20

15

10

-monomer

-dimer

-trimer

-tetramer

-sAPPα

-IgG light chain (25kda)

-IgG heavy chain (50kda)

-5mer

**B)**

**
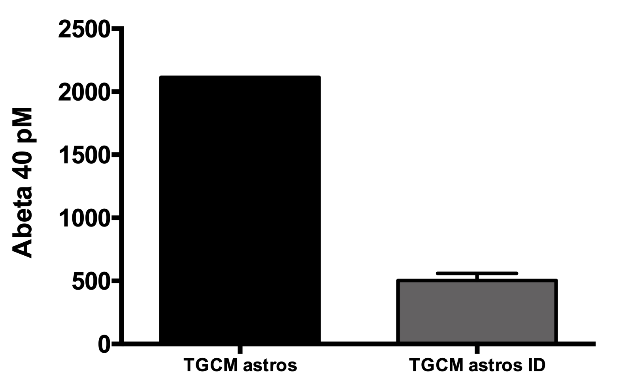

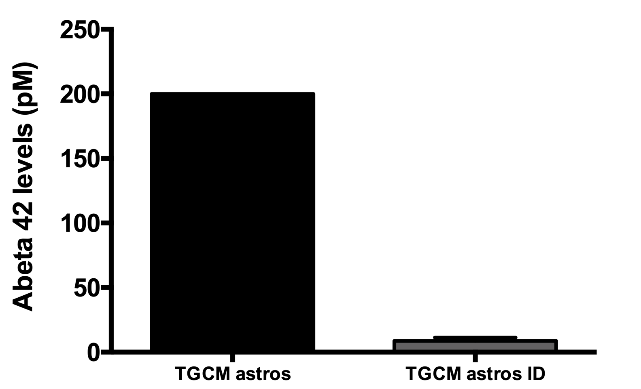
**

**A)** Aβ was depleted from TGCM and TGCM astro using 6E10. Briefly, culture medium was incubated with protein G Dynabeads (ThermoFisher) bound with 6E10 antibody (COVANCE) for 1 hour at 4°C. Beads were separated from medium using a magnetic stand to remove Aβ-6E10 complexes. Samples of medium before (INPUT) and after immunodepletion (IP) were immunoblotted with the 6E10 antibody against Aβ following concentration by immunoprecipitation (IP), showing efficient depletion of Aβ monomers and other Aβ species. Controls include TGCM or TGCM astro incubated with Dynabeads that were not coupled to 6E10 (TGCM + beads, TGCM astro + beads). Non-specific IgG bands are indicated in blue. **B)** Confirmation of Aβ immunodepletion by ELISA. Aβ levels in medium were determined using sandwich ELISA kits (ThermoFisher Scientific) as described in Wu et al. (2010)^1^.

**Additional Figure S2: Measures of neuronal health and complexity in mouse neurons challenged with WTCM astro, TGCM astro and TGCM astro-ID.**

**A)**


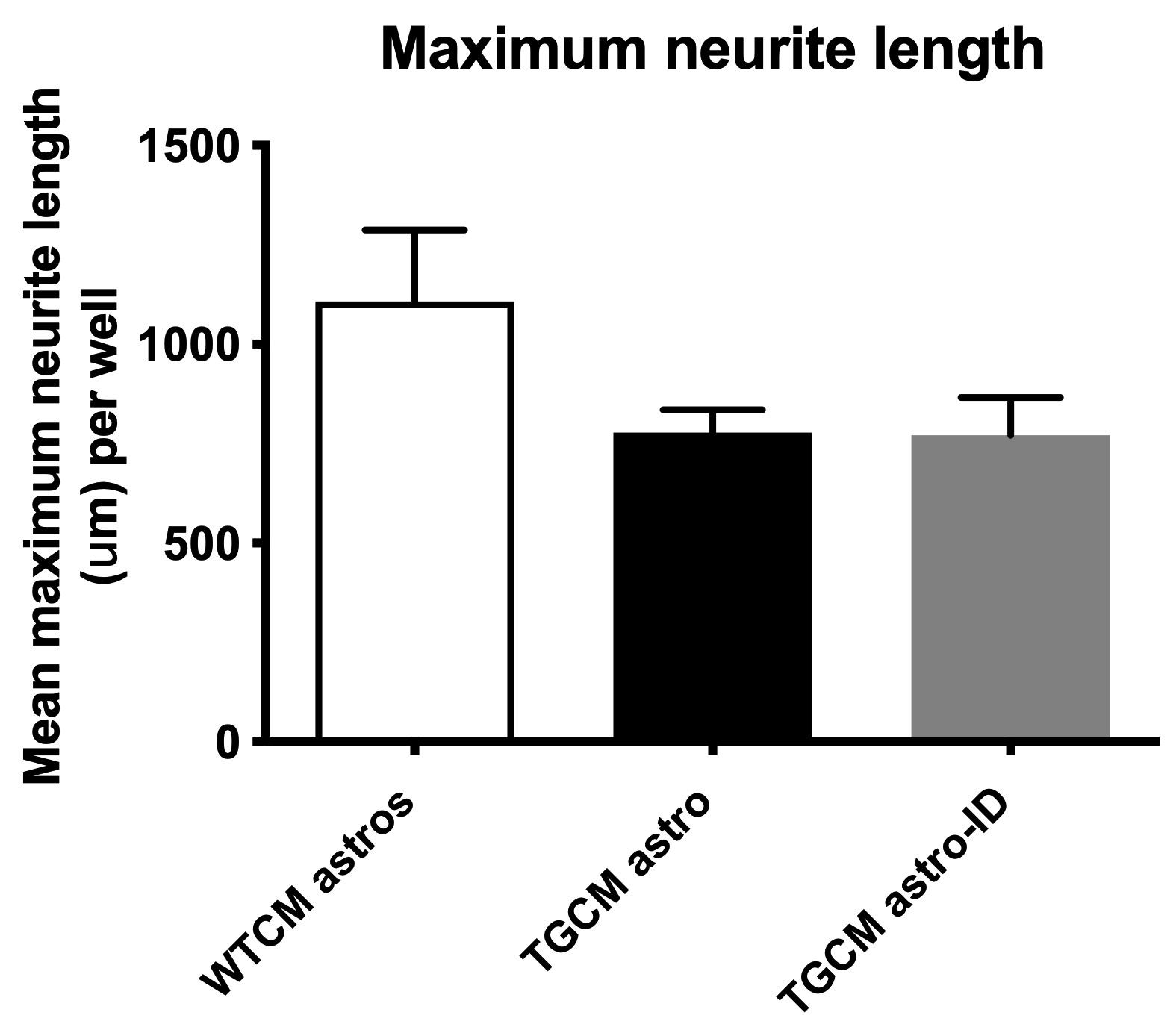

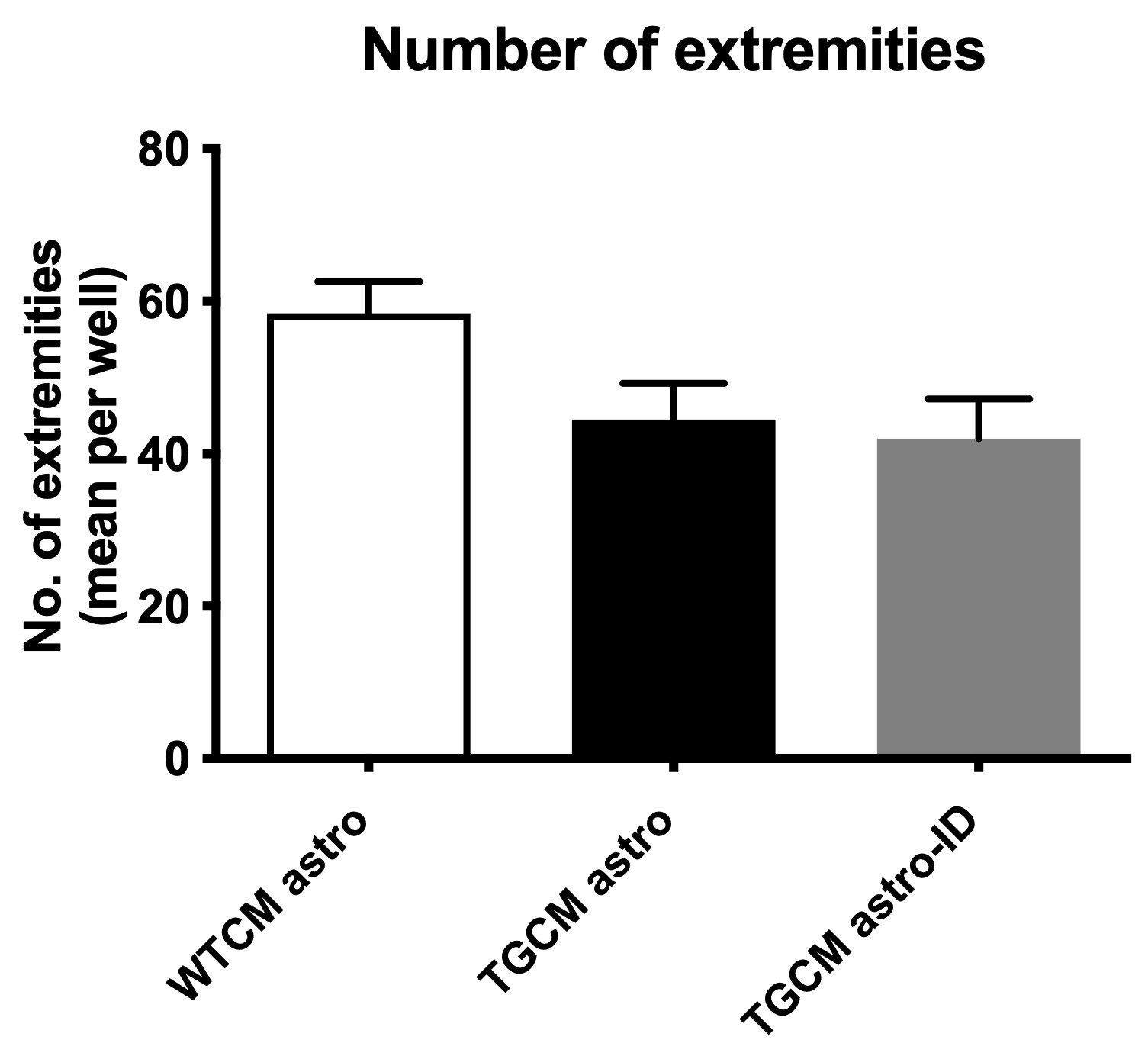

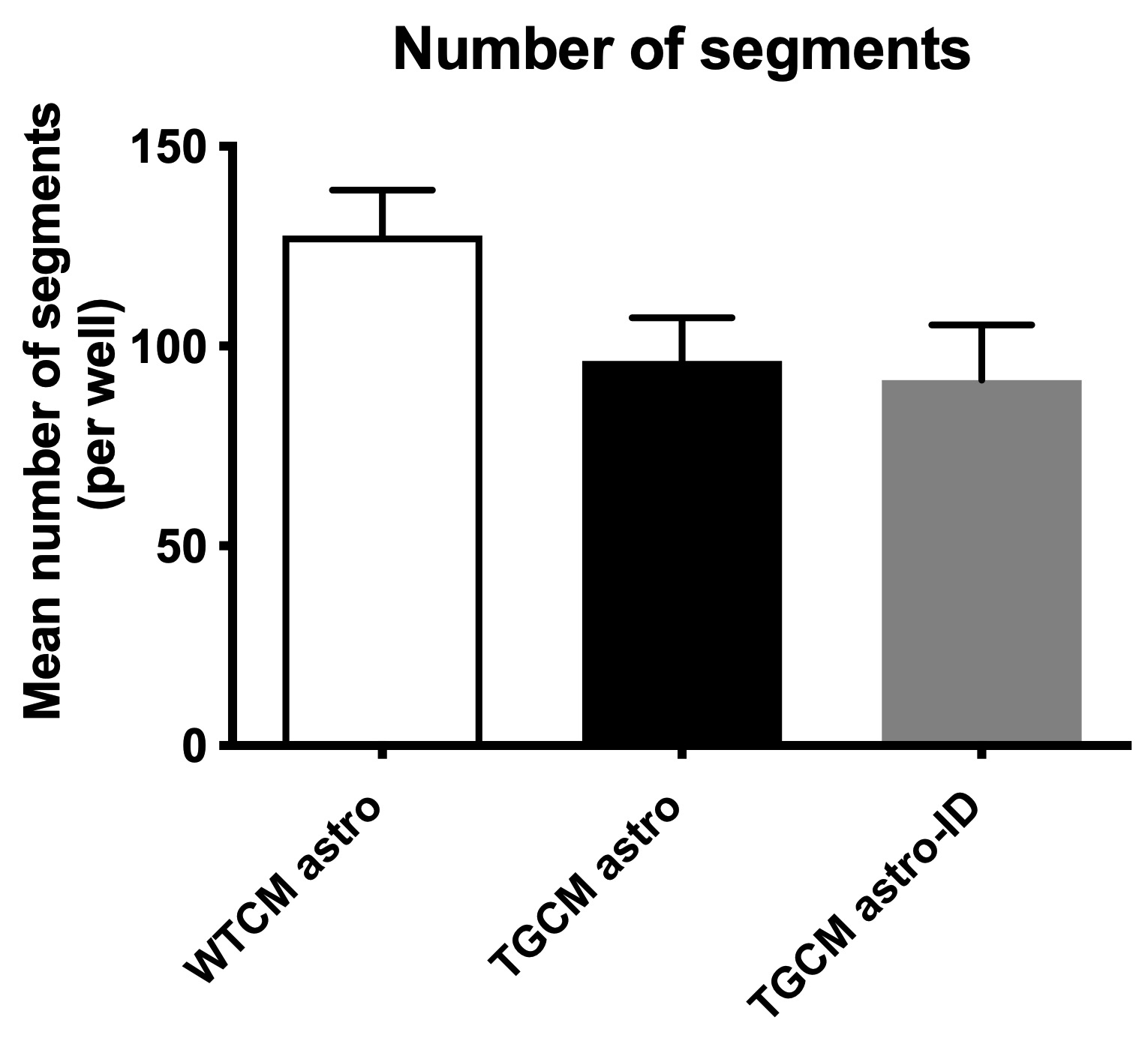


**
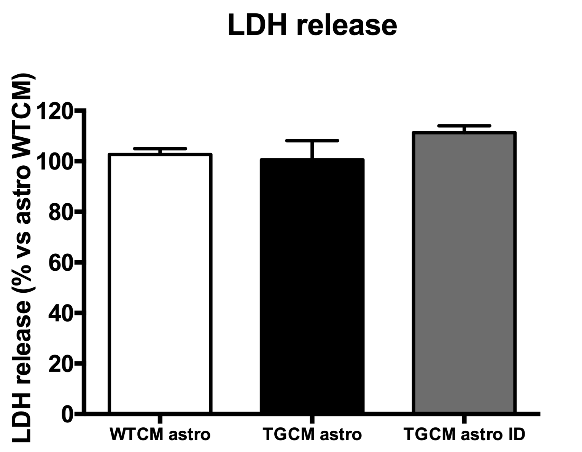
B)**

**A)** Conditioned medium was collected from primary cortical neurons from wild-type or Tg2576 mice (WTCM; TGCM) and applied to mouse astrocytes. The conditioned medium from stimulated astrocytes was collected, (WTCM astro; TGCM astro) and in some experiments Aβ was immunodepleted from the medium of TGCM treated astrocytes (TGCM astro-ID). These media were added to naïve WT primary neurons that were transfected with GFP at 7DIV. Neuronal complexity as a measure of neuron health was analysed for all cells in three wells per experimental replicate using Harmony software when neurons were 14DIV. This showed non-significant reductions in maximum neurite length, number of extremities and segments following treatment with TGCM astro and TGCM astro-ID. Data are mean +/-SEM, (n=5). **B)** The amount of lactate dehydrogenase (LDH) in the media of cultured neurons was determined as a measure of neuron health, using an LDH Cytotoxicity Kit from Thermo Fisher Scientific, according to the manufacturer’s instructions. Data are shown as percentage relative to control (WTCM astro) and are represented as mean +/-SEM, (n=5).

**Additional Figure S3: Measures of neuronal health and complexity in LUHMES challenged with WTCM h-astro, TGCM h-astro and TGCM h-astro-ID.**


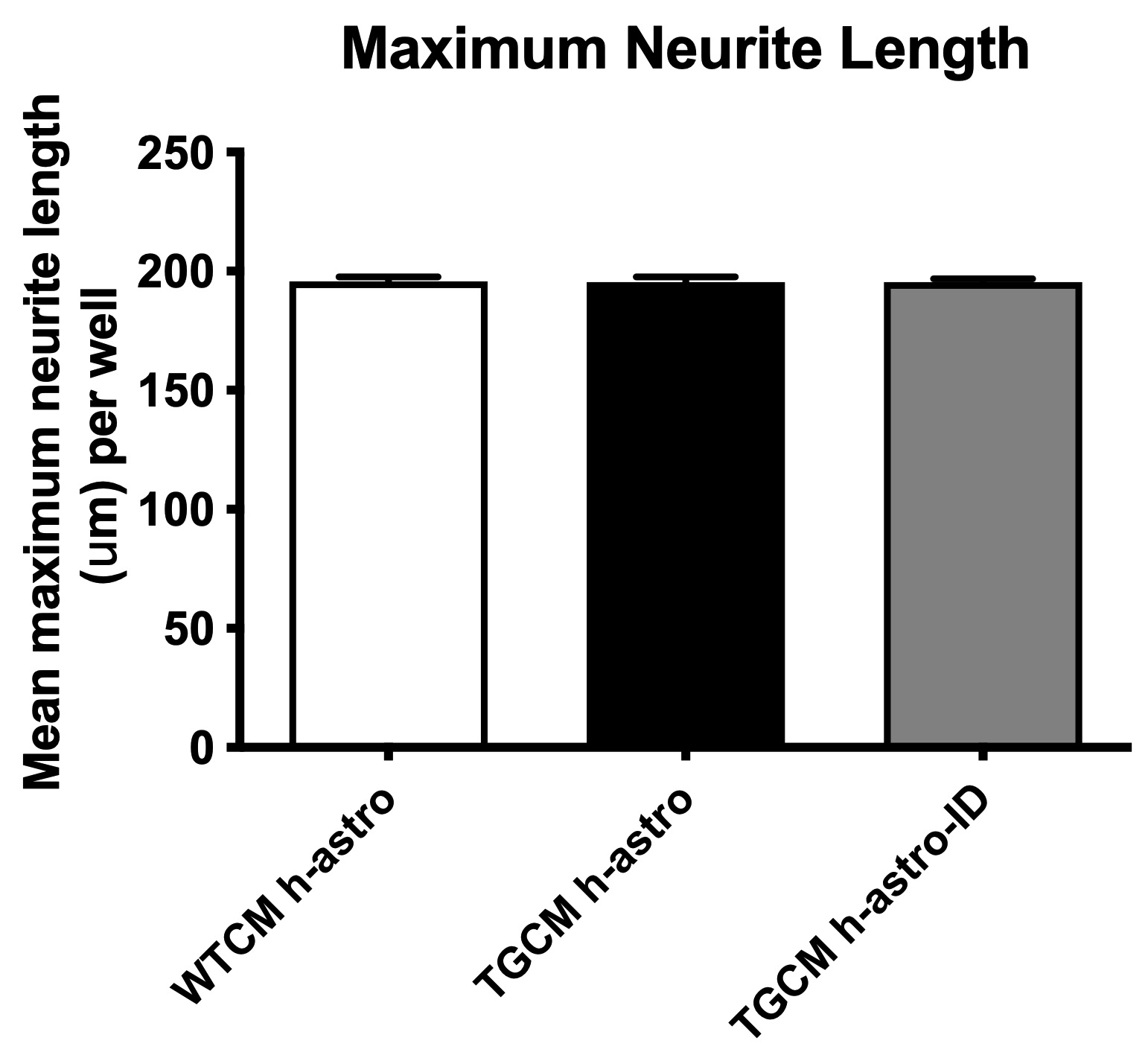

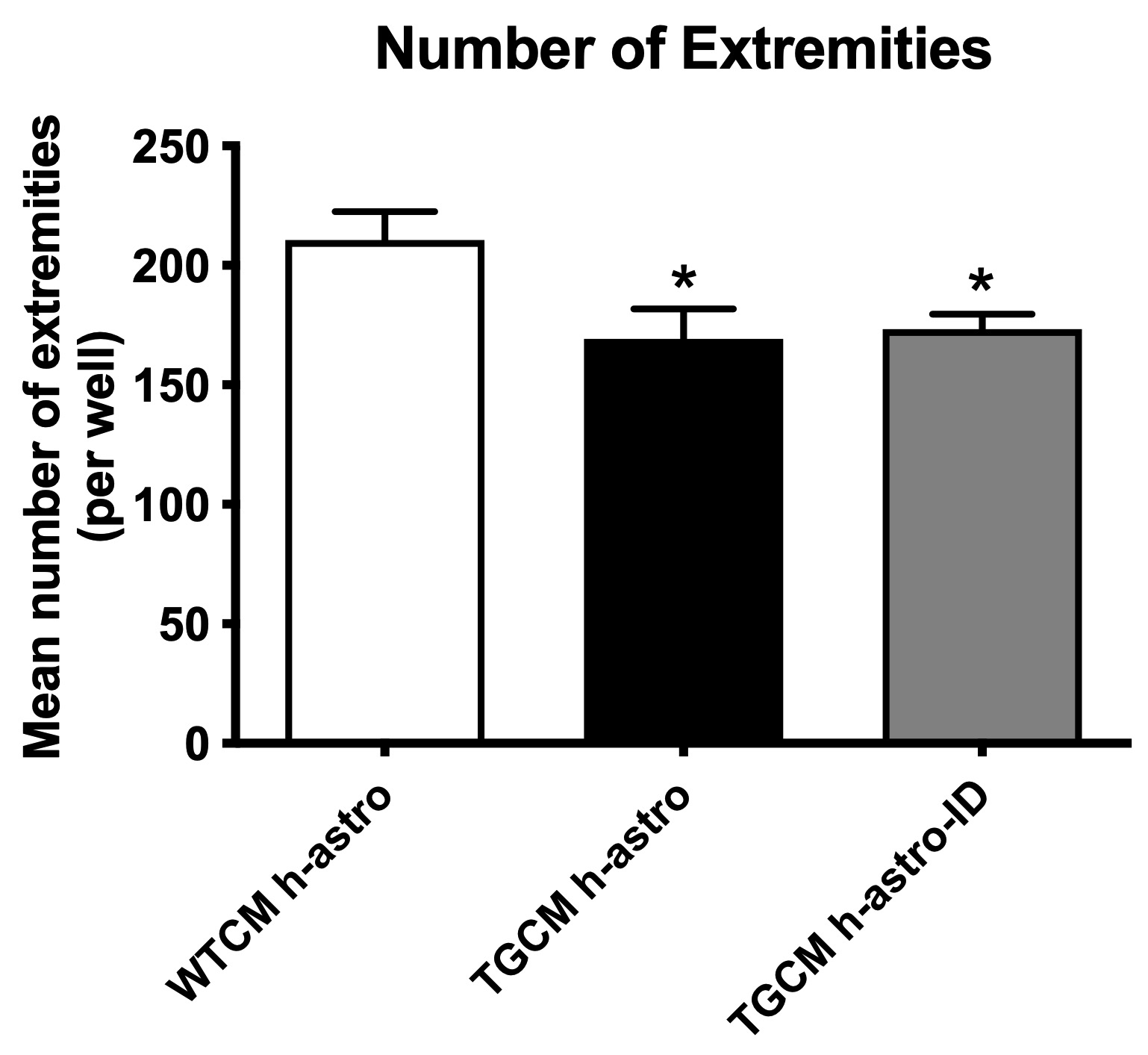

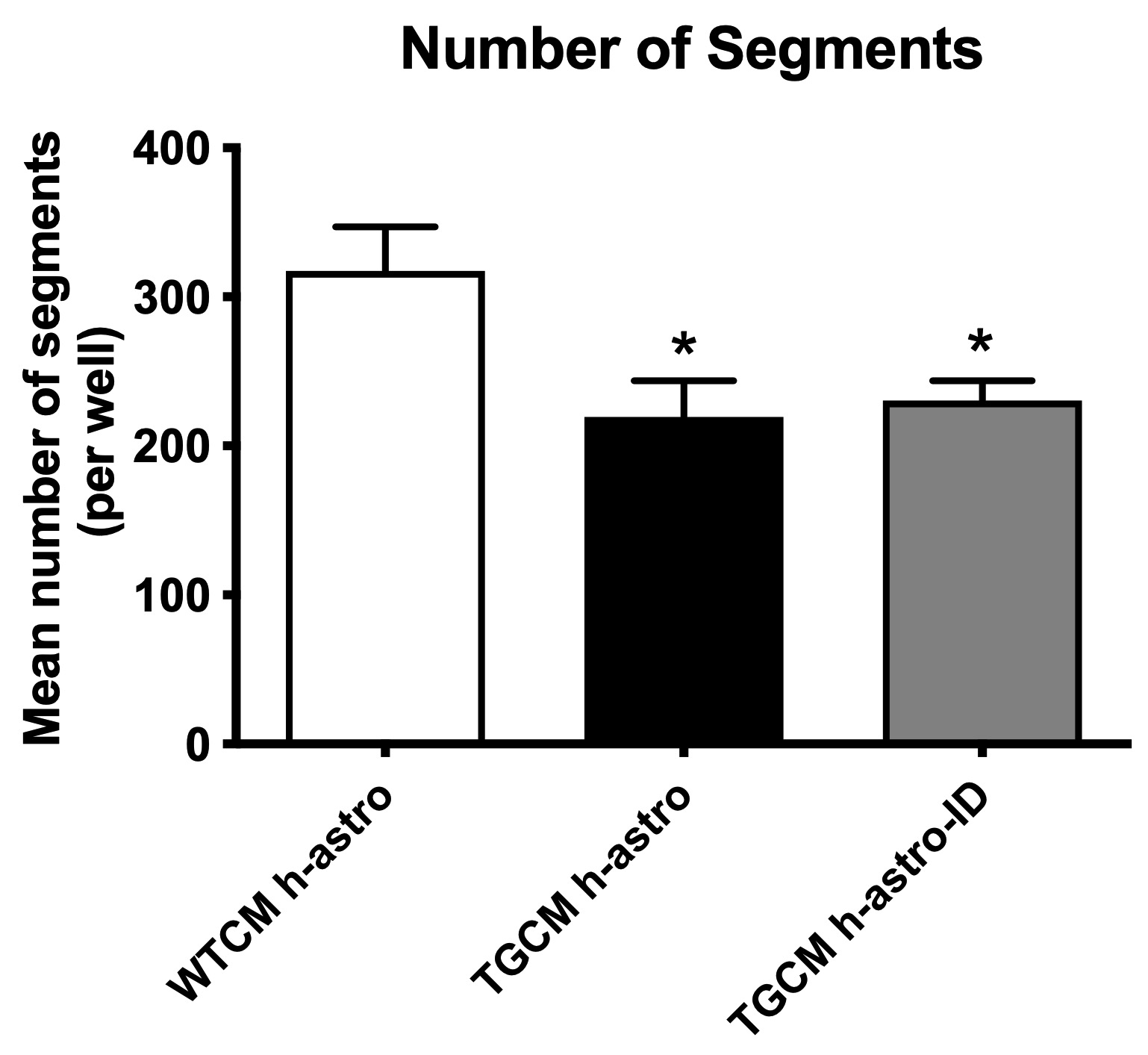


Conditioned medium was collected from primary cortical neurons from wild-type or Tg2576 mice (WTCM; TGCM) and applied to human iNPC-astrocytes. The conditioned medium from stimulated astrocytes was collected, (WTCM h-astro; TGCM h-astro) and in some experiments Aβ was immunodepleted from the medium of TGCM treated astrocytes (TGCM h-astro-ID). These media were added to differentiated LUHMES that had been transduced with GFP prior to differentiation. Neuronal complexity as a measure of neuron health was analysed for all cells with axons in three wells per experimental replicate using Harmony software. This showed small reductions in maximum neurite length (not significant), and significant reductions in the number of extremities and segments (p<0.05 for all) following treatment with TGCM h-astro and TGCM h-astro-ID (n=3). Data are mean +/-SEM.

**Additional Figure S4**

**Immunofluorescence of tau relative to MAP2 in neurons exposed to CXCL1.**

Neurons were fixed and immunolabelled with antibodies against MAP2 (green) and tau (red). Exposure of neurons to CXCL1 appeared to increase tau localisation in neurites (MAP2, green) relative to control conditions, and this was prevented by antagonism of neuronal CXCR2. Scale bar is 50μm.
